# Patient readmission for surgical wound infection

**DOI:** 10.1186/1753-6561-5-S6-P192

**Published:** 2011-06-29

**Authors:** LM Torres, RA Lacerda, R Turrini

**Affiliations:** 1 Enfermagem Médico-Cirúrgica, Escola de Enfermagem da Universidade de São Paulo, Nova Lima, Brazil; 2 Enfermagem Médico-Cirúrgica, Escola de Enfermagem da Universidade de São Paulo, São Paulo, Brazil

## Introduction / objectives

Surgical site infection (SSI) rates are underestimated mainly in the absence of a successful program of post discharge surveillance. Readmissions monitoring can contribute to accurate infection rates.

## Methods

Exploratory descriptive study, developed in a governmental hospital of tertiary care in Minas Gerais (Brazil), from January 2008 to December 2009. Medical records and reports of control infection practitioner of 98 patients readmitted with SSI were reviewed and the data were analyzed in relation to gender, age, co morbidities, length of staying, surgery, specialty, type of procedures, wound class, duration of surgery, SSI and micro-organisms.

## Results

Readmissions occurred in patients who underwent clean and potentially contaminated surgical procedures, with co morbidities commonly among people 50 years or older. Duration of surgery did not differ from the cut point recommended by CDC. *Staphylococcus aureus* predominated in orthopedic procedures and *Escherichia coli* in general surgery, both with multi-resistance profile below the results presented in other studies.

## Conclusion

Whereas the SSI occurred more frequently in clean surgeries and readmissions can provide information about the quality of care, these findings are important to control infection practitioner review the antibiotic prophylaxis protocols and surgical practices in patients undergoing clean and potentially contaminated procedures.

## Disclosure of interest

None declared.

